# Pre-pregnancy gene expression signatures are associated with subsequent improvement/worsening of rheumatoid arthritis during pregnancy

**DOI:** 10.1186/s13075-023-03169-6

**Published:** 2023-10-04

**Authors:** Matthew Wright, Mette Kiel Smed, J. Lee Nelson, Jørn Olsen, Merete Lund Hetland, Nicholas P. Jewell, Vibeke Zoffmann, Damini Jawaheer

**Affiliations:** 1 Children’s Hospital Oakland Research Institute, Oakland, CA USA; 2 Northwestern University, Feinberg School of Medicine, Chicago, IL USA; 3 Juliane Marie Centeret, Rigshospitalet, Copenhagen, Denmark; 4 Fred Hutchinson Cancer Center, Seattle, WA USA; 5 University of Washington, Seattle, WA USA; 6 University of California Los Angeles, Los Angeles, CA USA; 7 Aarhus University Hospital, Aarhus, Denmark; 8 DANBIO Registry and Copenhagen Centre for Arthritis Research, Centre for Rheumatology and Spine Diseases, Rigshospitalet, Glostrup, Denmark; 9 University of Copenhagen, Copenhagen, Denmark; 10 London School of Hygiene and Tropical Medicine, London, UK; 11 Division of Rheumatology, Northwestern University, Feinberg School of Medicine, Chicago, IL USA

**Keywords:** Rheumatoid arthritis, Pregnancy, Pre-pregnancy biomarkers, Gene expression, RNA sequencing (RNA-seq), Co-expression network analysis

## Abstract

**Background:**

While many women with rheumatoid arthritis (RA) improve during pregnancy and others worsen, there are no biomarkers to predict this improvement or worsening. In our unique RA pregnancy cohort that includes a pre-pregnancy baseline, we have examined pre-pregnancy gene co-expression networks to identify differences between women with RA who subsequently improve during pregnancy and those who worsen.

**Methods:**

Blood samples were collected before pregnancy (T0) from 19 women with RA and 13 healthy women enrolled in our prospective pregnancy cohort. RA improvement/worsening between T0 and 3rd trimester was assessed by changes in the Clinical Disease Activity Index (CDAI). Pre-pregnancy expression profiles were examined by RNA sequencing and differential gene expression analysis. Weighted gene co-expression network analysis (WGCNA) was used to identify co-expression modules correlated with the improvement/worsening of RA during pregnancy and to assess their functional relevance.

**Results:**

Of the 19 women with RA, 14 improved during pregnancy (RA_improved_) while 5 worsened (RA_worsened_). At the T0 baseline, however, the mean CDAI was similar between the two groups. WGCNA identified one co-expression module related to B cell function that was significantly correlated with the worsening of RA during pregnancy and was significantly enriched in genes differentially expressed between the RA_improved_ and RA_worsened_ groups. A neutrophil-related expression signature was also identified in the RA_improved_ group at the T0 baseline.

**Conclusion:**

The pre-pregnancy gene expression signatures identified represent potential biomarkers to predict the subsequent improvement/worsening of RA during pregnancy, which has important implications for the personalized treatment of RA during pregnancy.

**Supplementary Information:**

The online version contains supplementary material available at 10.1186/s13075-023-03169-6.

## Background

It is well established that rheumatoid arthritis (RA), though incurable, can improve naturally during pregnancy in a substantial proportion (50–75%) of women and that it may worsen or remain unchanged in others [1, 2]. Unfortunately, thus far, no biomarkers have been identified that can predict, at the pre-pregnancy stage, whether a woman with RA will subsequently improve or worsen during pregnancy. Consequently, women with RA who are considering a pregnancy and do not wish to take medications during their pregnancy, are often concerned about whether their disease will worsen during the pregnancy if they choose to stop their medications.

We had previously reported that, at the pre-pregnancy baseline in our unique prospective pregnancy cohort, women with RA who subsequently improved during pregnancy (RA_improved_) and those who worsened (RA_worsened_) exhibited different RA-associated expression signatures when compared to healthy women [3]. We have now built on those differential gene expression findings in a larger sample of our cohort, using co-expression network analysis [4] as a systems-based approach to dissect the complexity of our pre-pregnancy transcriptome data. Thus, in this hypothesis-generating study, we aimed to build densely connected sub-networks (modules) of genes with highly correlated expression and to determine whether any of these modular repertoires were correlated with our trait of interest, namely the improvement/worsening of RA during pregnancy. We sought to assess whether such a module would be enriched in genes that exhibited significant differential expression between the RA_improved_ and RA_worsened_ women. Furthermore, since genes co-expressed within a module tend to be functionally related and co-regulated [5], we performed functional analysis of modules to gain insight into the underlying biological differences between the RA_improved_ and RA_worsened_ women at the pre-pregnancy stage.

## Subjects and methods

### Study subjects

Healthy women and women with RA of Danish descent who were planning a pregnancy were enrolled in our pregnancy cohort in Denmark and were prospectively followed, as previously described [6, 7]. A set of 19 women with RA and 13 healthy women from this cohort were included in the present study; these included the 9 RA and 5 healthy women from our previous study [3]. The study was approved by the Ethics Committee for Region Hovedstaden (Protocol #: H-2–2009-150) and the Danish Data Protection Agency (Data processing ID: RH-2015–02; record #: i-suite 03601) in Denmark, the Children’s Hospital Oakland Research Institute Institutional Review Board (IRB number: 2009–073), and the Northwestern University IRB (IRB number: STU00217093). All subjects provided written informed consent prior to enrollment.

### Assessment of RA disease activity

RA disease activity was assessed using the Clinical Disease Activity Index (CDAI) [8], because it does not include acute phase reactants such as C-reactive protein (CRP) whose levels are known to fluctuate during pregnancy. The change in CDAI (ΔCDAI) from before pregnancy (T0) to the pregnancy time point where improvement/worsening was maximal (second trimester (T2) for 2 women who improved and third trimester (T3) for all others) was used to determine whether disease activity improved or worsened. Patients were categorized as having improved during pregnancy (RA_improved_), if their ΔCDAI fit the criteria for a minimum clinically important difference (MCID) based on baseline (T0) disease activity; ΔCDAI values of 12, 6, and 1 were used as a threshold when disease activity at T0 was high, moderate, or low, respectively [9]. Those women with an increase in CDAI from T0 to T3, satisfying the MCID criteria for worsening of disease activity, were included in the “worsened” subset, referred to as RA_worsened_.

### Sample collection, processing, and RNA sequencing

Only pre-pregnancy samples were used in the present study. Blood samples were drawn into PAXgene tubes and frozen. Total RNA was manually extracted using the PAXgene Blood RNA Kit according to the manufacturer’s protocol, and RNA integrity was assayed using a 2100 Bioanalyzer. 250 ng of total RNA were first depleted of ribosomal RNAs and globin mRNAs using the KAPA RiboErase kit (Roche) and KAPA globin depletion hybridizing oligos (Roche), respectively. Barcoded and stranded cDNA libraries prepared using the KAPA RNA HyperPrep kit were pooled and sequenced on an Illumina NovaSeq 6000 instrument, targeting an average of 100 million 150 bp paired-end reads.

### Bioinformatics analysis

Rigorous quality control (QC) of the raw data was performed using FASTQC, Picard, and HTSTREAM. Raw FASTQ reads were trimmed using Cutadapt (v2.4) and aligned to the human genome (GRCh38; Ensembl v98) using HISAT2 (v 2.1.0). Multi-mapped reads were filtered using Samtools. Aligned reads were assembled into transcripts and merged using StringTie (v 2.1.1).

Novel lncRNAs in our assembled transcripts were assessed by removing transcripts that (1) overlapped with any known transcript on the same strand (Bedtools v 2.28.0); (2) had open reading frames (ORFs) > 100 amino acids (TransDecoder v 5.5.0); (3) when translated, had similarity to known proteins/protein domains [blastx hits to the RefSeq Protein or Pfam databases] (Blast + v 2.7.1, flags: -strand plus -max_target_seqs 1 -evalue 1E−5); (4) classified as coding by at least one of 3 tools for detecting coding potential [CPAT (v 3.0.2), CPC (v 2.0), and FEELnc (v 0.2)]; and (5) were single-exon. The remaining transcripts were classified as “novel lncRNAs” and appended to the Ensembl v98 gtf file. The resulting annotation file (gtf) containing all known and newly discovered (from our data) transcripts was used as a reference to obtain gene-level counts for all known genes and lncRNAs as well as novel lncRNAs with featureCounts (v 2.0.0, flags: -s 2 -p).

Raw counts were loaded into R, and “rRNA” and “pseudogene” gene types were removed, along with gene types “misc_RNA,” “Mt_tRNA,” “scaRNA,” “snRNA,” “snoRNA,” and “TEC.” Low-expression genes were removed by keeping only genes with CPM > 10/L in 6 or more samples, where *L* is the minimum library size in millions. Library size was normalized in edgeR (v 3.30.3) with the trimmed mean of *M*-values (TMM) method using the calcNormFactors function and normalized counts were exported for downstream statistical analyses.

### Deconvolution of bulk RNA-seq data

To estimate cell type proportions in each sample, raw reads were aligned to the Ensembl v98 transcriptome using kallisto (v 0.46.1) and aggregated to gene-level data using tximport (v 1.18) in R. Gene-level data for each sample were deconvolved using CIBERSORTx and the accompanying LM22 signature matrix that is based on 22 human immune cell types [10]. We used principal components analysis (PCA) to condense the information about changes in all 22 estimated cell type proportions into principal components (PCs) as proposed by Kong et. al [11] and tested them for association with gene expression.

### Differential gene expression analysis

To compare normalized T0 gene-level counts between the groups (RA_improved_ vs RA_worsened_; RA_improved_ or RA_worsened_ vs healthy), differential gene expression analysis was performed using generalized linear model (GLM) likelihood ratio tests and the contrast argument of the *glmLRT* function in edgeR. Correction for multiple testing was performed using the false discovery rate (FDR) method. An FDR value threshold of 0.05, in combination with a fold change (FC) of at least 1.5, was used to assess significance.

### Co-expression network analysis

Co-expression analysis of normalized gene-level counts from the RA_improved_, RA_worsened_, and healthy women was performed in R using the weighted gene co-expression network analysis (WGCNA) package (v1.69) [12]. The following specifications were used: power = 5, networkType = signedHybrid, corType = bicor, maxPOutliers = 0.1, and mergeCutHeight = 0.25. Each module was examined for (i) correlation with the clinical trait of improvement/worsening during pregnancy, (ii) the presence of genes that were differentially expressed (DE) between the RA_improved_ and RA_worsened_ groups at T0 and enrichment of DE genes, and (iii) hub genes, by selecting the top 10% genes based on network adjacency. Furthermore, since genes co-expressed within a module tend to be co-regulated and functionally related, functional analysis of the modules was performed to gain insight into the potential functions of the genes and lncRNAs being co-expressed within specific modules. Enrichment of genes differentially expressed between the RA_improved_ and RA_worsened_ groups within co-expression modules was assessed by a hypergeometric test. Protein–protein interaction (PPI) among the hub genes was examined using the Search Tool for the Retrieval of Interacting Genes (STRING) database [13].

### Functional enrichment

Enrichment of Gene Ontology (GO) terms and KEGG and Reactome pathways were assessed using WebgestaltR [14]. Interactions between proteins encoded by the significant genes were based on data from the STRING database [15] and visualized in Cytoscape (v3.8.1) [16].

### Transcription factor analysis

Enrichment of transcription factor targets was performed using the *fora* function in the fgsea R package [17]. Transcription factor-target regulons were pulled from the DoRothEA database, using confidence levels A, B, and C [18].

### Identification of B cell-related genes

Expression in B cells was based on expression patterns reported in The Human Protein Atlas [19] and the Immunological Genome (ImmGen) Project [20], as well as from a previous report of an 85-gene B cell signature identified from freshly isolated B cells [21].

## Results

### Study subjects

**Fig. 1:**
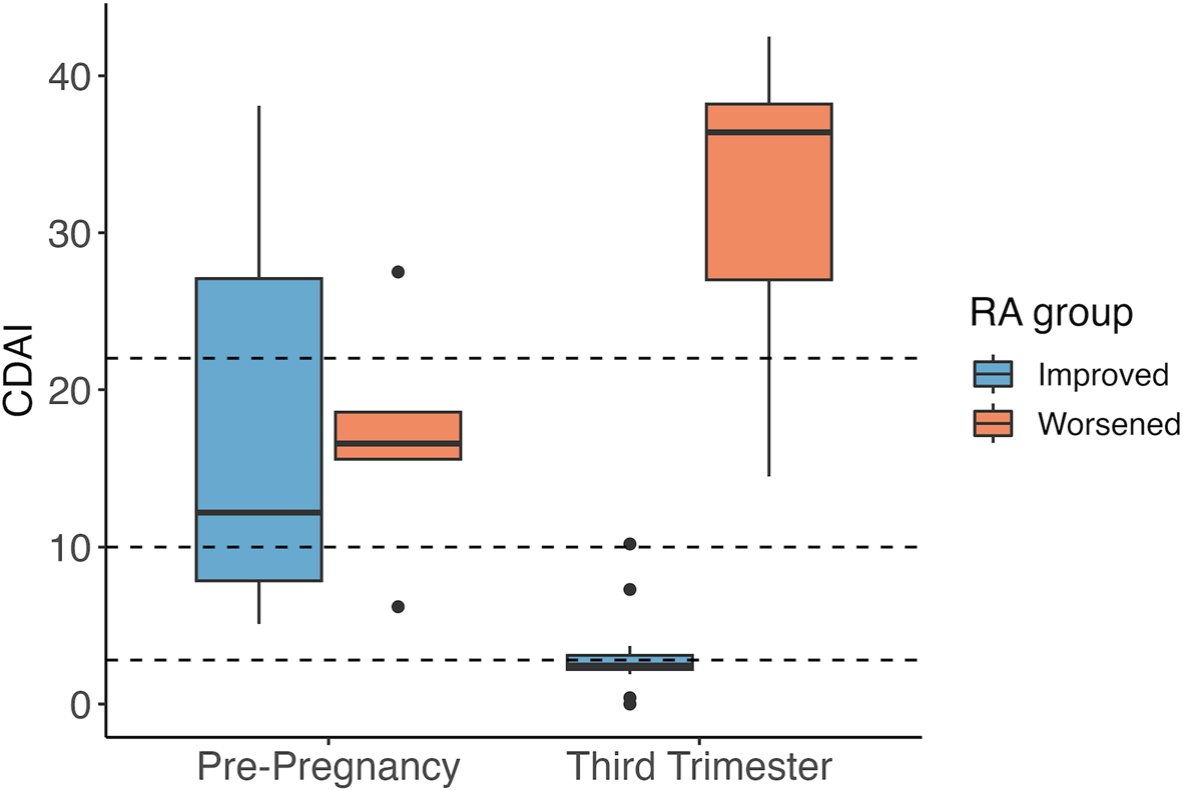
RA disease activity before and during pregnancy. At the pre-pregnancy (T0) baseline, the mean disease activity scores (CDAI) were similar between the 14 women with RA who subsequently improved during pregnancy (RA_improved_) and the 5 women who worsened (RA_worsened_)

Of the 19 women with RA, 14 subsequently improved during pregnancy (RA_improved_) while 5 worsened (RA_worsened_), based on MCID thresholds. Nevertheless, at the pre-pregnancy (T0) baseline, the mean CDAI was similar between the two groups [RA_improved_ (mean ± S.D.): 16.8 ± 11.5; RA_worsened_: 16.9 ± 7.6, *p* = 0.9] (Fig. 1). Other patient characteristics at the T0 baseline were also comparable between the two RA groups, including RA duration [RA_improved_: 7.6 ± 6.8 years; RA_worsened_: 8.5 ± 4.4 years, *p* = 0.4], and age at the T0 visit [RA_improved_: 31.0 ± 5.1 years; RA_worsened_: 33.2 ± 3.3 years, *p* = 0.4]. Characteristics of the healthy women have been described elsewhere [22]; one woman (of 14) was excluded due to missing pre-pregnancy data. The mean age of the healthy women at the T0 visit was 28.7 ± 3.7 years. The medications that the women with RA were taking at or before the T0 time point are summarized in Table 1.

**Table 1:** Medications taken at and before the pre-pregnancy visit. Within each of the RA_improved_ and RA_worsened_ groups, the number of women who did not take any medications before pregnancy is shown, together with the numbers who were on different medications. Some women had taken sulfasalazine, prednisolone, or methotrexate during the 3 months preceding the pre-pregnancy visit, but by the time of the pre-pregnancy visit, they had already stopped those medications

	**RA**_**improved**_ **(*n* = 14)**	**RA**_**worsened**_ **(*n* = 5)**
***No medications being taken at the pre-pregnancy baseline***	5^¶^	1
***Medications being taken at the pre-pregnancy baseline***		
Prednisolone + sulfasalazine	3^*^	
Prednisolone + sulfasalazine + etanercept		1
Infliximab or adalimumab		2^¶^
Prednisolone + infliximab	1	
Sulfasalazine	4^¥^	
Benepali	1	
Hydroxychloroquine + sulfasalazine		1^*^
***Medications taken 3 months prior to pre-pregnancy visit (but no longer taken by the time of that visit)***		
Sulfasalazine (^¶^)	1	1
Methotrexate (^*^)	1	1
Prednisolone (^¥^)	1	

### Data QC

After rigorous QC, the gene expression (RNA-seq) data were visualized on a PCA plot (Additional file 1: Fig. S1). There was a small degree of overlap between the RA_improved_ and RA_worsened_ clusters, and the healthy women cluster was mostly within that area of overlap.

### Differences in cell type proportions between the 2 RA groups at T0

Deconvolution of the RNA-seq data using CIBERSORTx produced relative cell type proportions, limited to the 22 immune cell types present in the LM22 reference panel [10]. No significant differences in these cell type proportions were observed between the RA_improved_ and RA_worsened_ groups at T0 (Additional file 2: Fig. S2).

### Differential gene expression

**Fig. 2:**
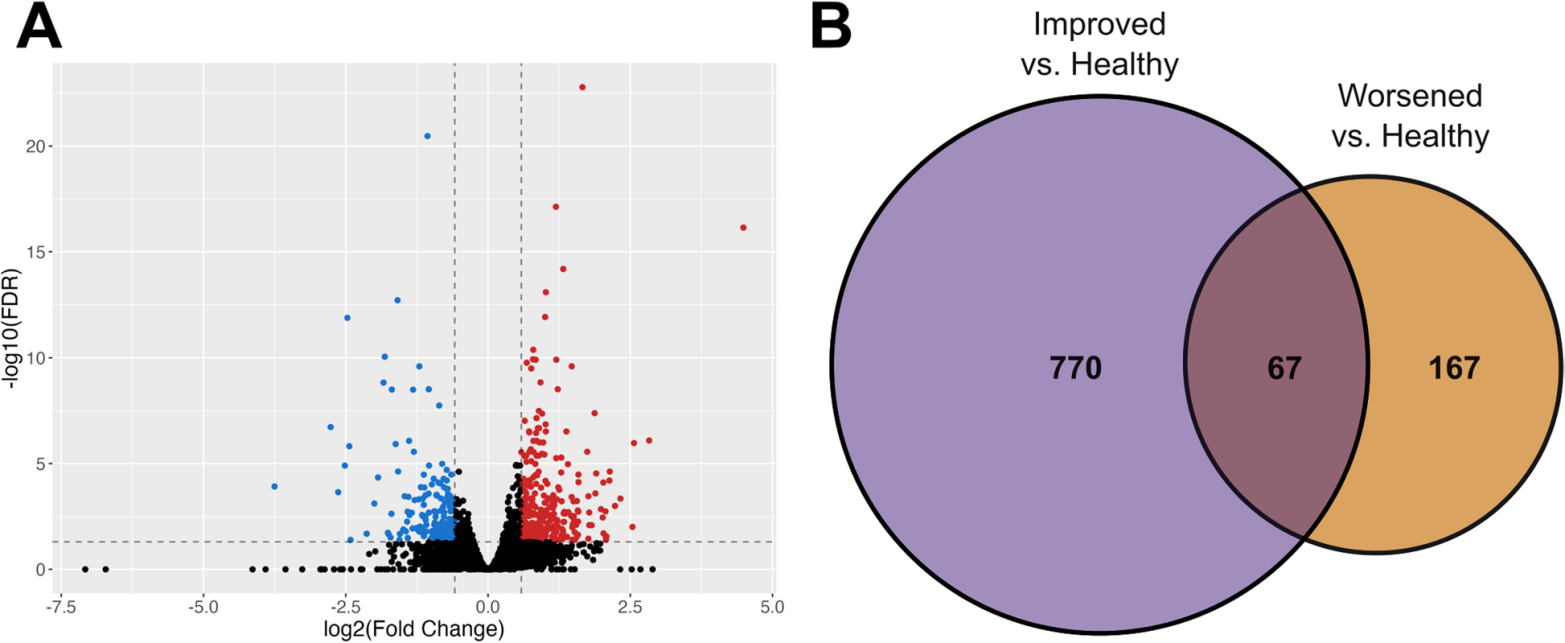
RA gene expression signatures at the pre-pregnancy baseline. **A** A total of 448 protein-coding genes and 137 lncRNAs were differentially expressed (FDR < 0.05, fold change [FC] ≥ 1.5) between the RA_improved_ and RA_worsened_ women at the pre-pregnancy baseline, as shown in the volcano plot. **B** When T0 expression profiles of each of the RA groups were compared to that of the same 13 healthy women, the RA-associated gene expression signatures observed showed little overlap with each other. Only 42 coding genes, 24 lncRNAs, and 1 miRNA were common to both “RA signatures”

At T0, 448 protein-coding genes and 137 lncRNAs were differentially expressed (FDR < 0.05, fold change [FC] ≥ 1.5) between the RA_improved_ and RA_worsened_ women (Fig. 2A). The protein-coding genes were enriched among GO terms such as myeloid leukocyte migration (FDR = 0.003), innate immune response (FDR = 0.003), regulation of leukocyte chemotaxis (FDR = 0.01), and B cell proliferation (FDR = 0.01). Furthermore, some neutrophil-related genes (SERPINB10, CAMP, CXCL6, MMP9, PADI4, NECAB2) were over-expressed (FC: 1.9–3.4) among the RA_improved_ women. Numerous B cell-related genes (such as CD19, CD22, CD40, CD72, CD79A, BLNK, IL7, MS4A1, PAX5, BLK, and several immunoglobulin heavy chain genes) were over-expressed (FC: 1.5–4.1) among the RA_worsened_ women. The differential expression output for all genes analyzed (*n* = 19,468) is provided in Additional file 3: Table S1.

When each RA group was compared to the healthy women [RA_improved_ (or RA_worsened_) vs healthy], the genes with RA-associated expression (RA_improved_: 545 coding genes, 276 lncRNAs, and 16 miRNAs; RA_worsened_: 160 coding genes, 73 lncRNAs, and 1 miRNA; FDR < 0.05, FC ≥ 1.5) were mostly specific to each group, with little overlap between them (42 coding genes and 24 lncRNAs, 1 miRNA) (Fig. 2B). As was observed in the results of the RA_improved_ vs RA_worsened_ analysis, an over-expressed neutrophil signature (FC: 1.5–5.6) was apparent in the RA_improved_ group (vs healthy), enriched in genes involved in inflammatory response (FDR = 1.6E−11), neutrophil-mediated immunity (FDR = 2.7E−10), and myeloid cell activation involved in immune response (FDR = 2.7E−10), among others. On the other hand, the differentially expressed genes in the RA_worsened_ group included an over-expressed B cell signature (FC: 1.5–2.4), although, overall, the differentially expressed genes were not enriched within any specific GO terms.

### Co-expression network analysis

**Fig. 3:**
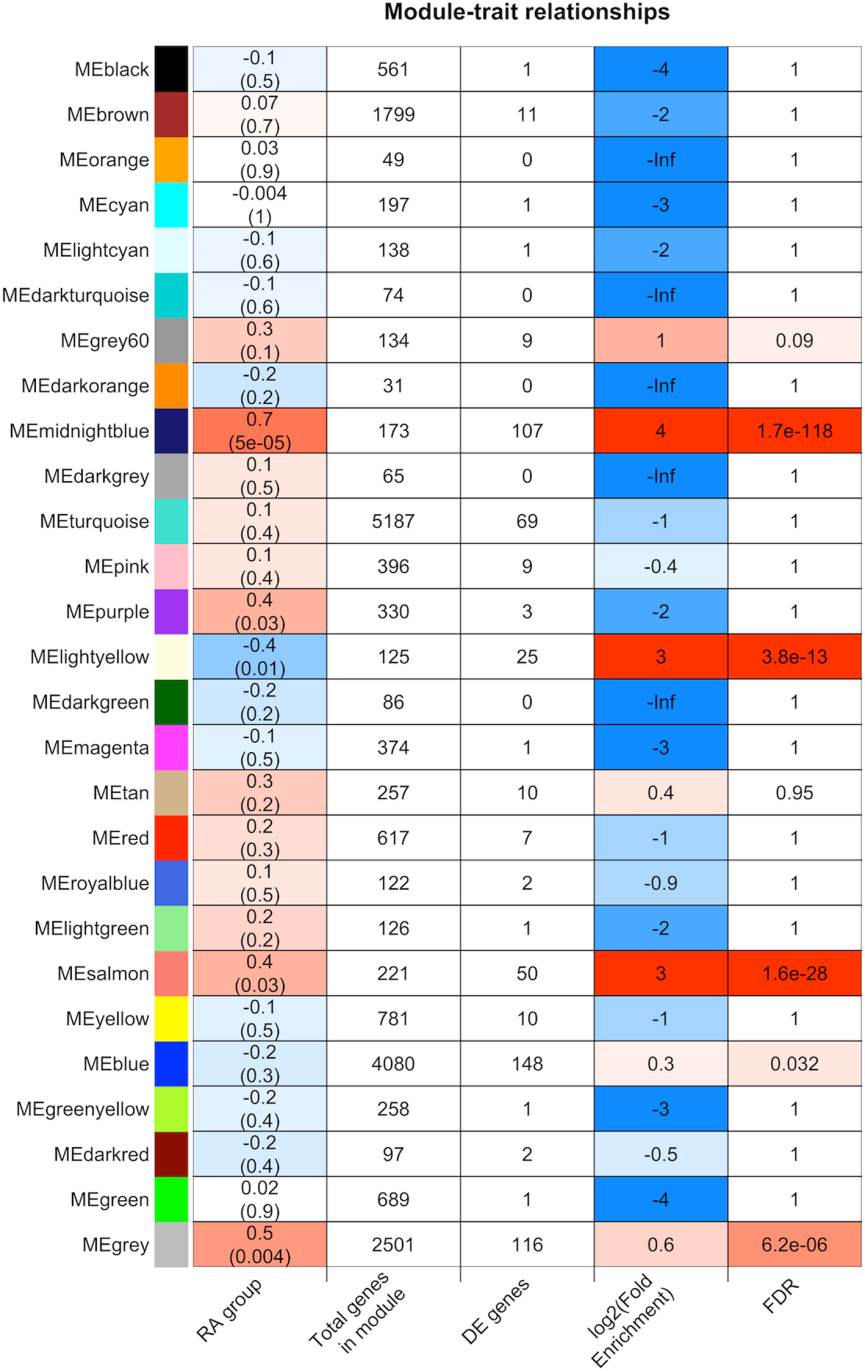
Co-expression of protein-coding genes and long non-coding RNAs within functional modules identified by WGCNA at the pre-pregnancy baseline. Using the gene-level counts from the RA_improved_, RA_worsened_ and healthy women at the pre-pregnancy baseline, weighted gene co-expression network analysis (WGCNA) identified 27 modules of coding genes and lncRNAs with highly correlated intra-module expression. The different modules (with color labels) are shown on the left. For each module, the five columns to the right indicate, in respective order, (1) the correlation between module eigengene expression and subsequent improvement/worsening during pregnancy (RA group), (2) total number of genes (among all genes analyzed) that clustered within the module, (3) the number of genes (coding/lncRNAs) differentially expressed between the two RA groups that are co-expressed within the module, (4) fold enrichment of these differentially expressed genes within the module, and (5) the FDR value for the enrichment analysis. The midnightblue, light yellow, and salmon modules were significantly enriched in genes differentially expressed between the RA_improved_ and RA_worsened_ groups, and their eigengene expression patterns were significantly correlated with subsequent improvement or worsening of RA during pregnancy. The grey module represents genes that were not co-expressed and were not assigned to any of the co-expression modules

WGCNA identified 27 modules or sub-networks of coding genes and lncRNAs with tightly correlated intra-module expression (Fig. 3). Of these, 3 modules (midnightblue, light yellow, and salmon) were significantly enriched in genes that were differentially expressed between the RA_improved_ and RA_worsened_ groups, and the eigengene expression patterns within these modules were also significantly correlated with the clinical trait of interest, i.e., subsequent improvement or worsening during pregnancy (midnightblue: *r* = 0.65; *p* = 5E−05; light yellow: *r* =  − 0.45, *p* = 0.01; salmon: *r* = 0.38, *p* = 0.03).

#### The midnightblue module

The midnightblue module consisted of 173 members that were tightly co-expressed, 107 (62%) of which (84 protein-coding genes, 21 lncRNAs, and 2 miRNAs) were differentially expressed between the RA_improved_ and RA_worsened_ groups. Thus, there was a significant enrichment (2^4.4, i.e., 21-fold, FDR = 1.7E−118) of the differentially expressed genes within this module (Fig. 3). The 84 protein-coding genes included numerous B cell-related genes (such as BLK, BLNK, CD19, CD22, CD72, CD79A, CD79B, CD180, CXCR5, FCRLA, FCRL1, FCRL2, LARGE1, MS4A1, PAX5, TNFRSF13B, TNFRSF13C, and some immunoglobulin heavy chain genes), all of which were over-expressed in RA_worsened_ by 1.5- to 2.8-fold; the 21 lncRNAs included FAM30A and COPDA1, both of which have been implicated in B cell regulation [both over-expressed in RA_worsened_ by 1.7- and 3.7-fold, respectively]; the 2 miRNAs, MIR4537 and MIR4539, were 1.6- and 1.7-fold over-expressed in RA_worsened_ group, respectively.

**Fig. 4:**
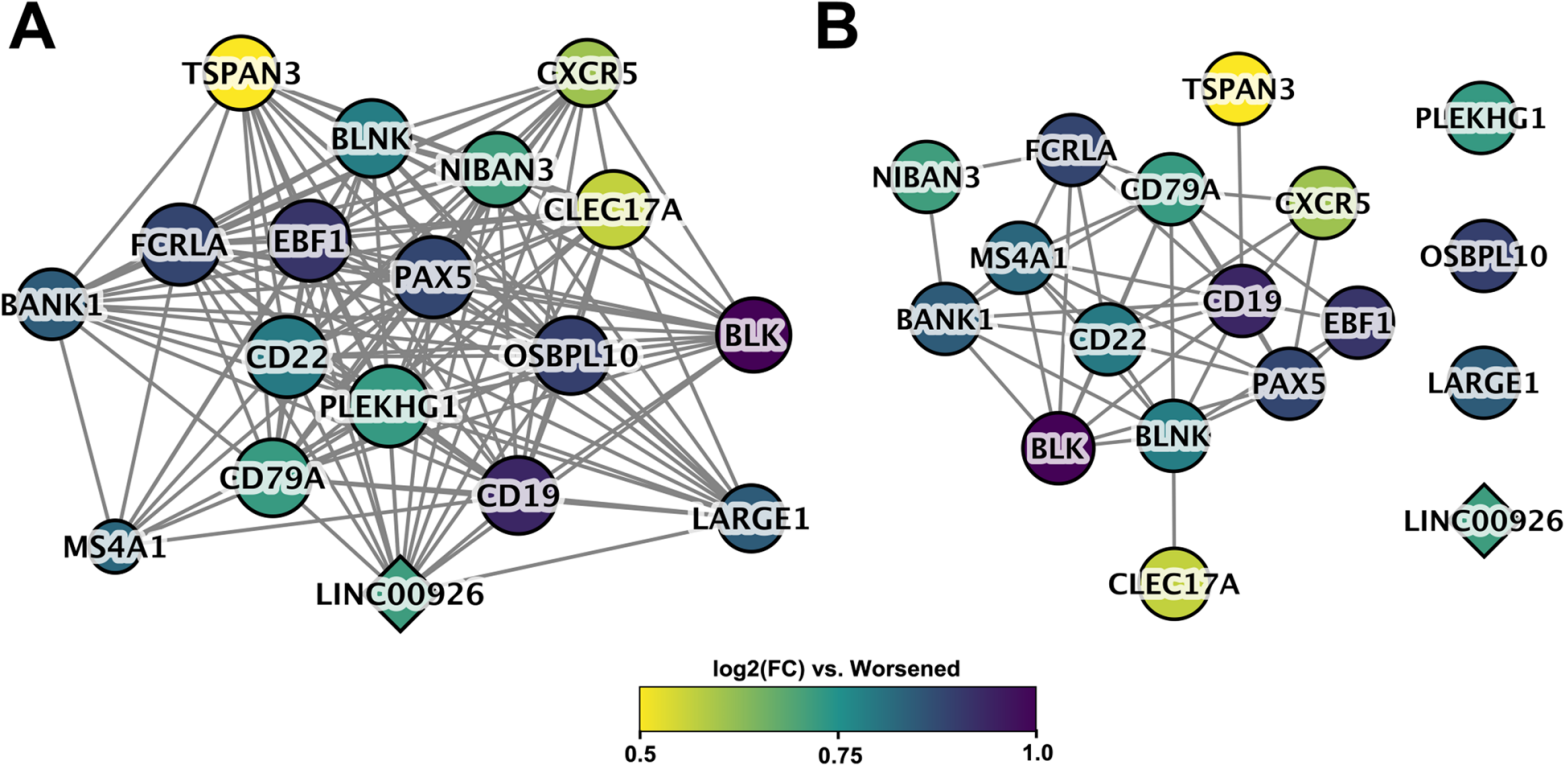
Midnightblue module hub genes. **A** A total of 18 hub genes were identified within the midnightblue module, by selecting the top 10% of genes having the highest degree of intra-modular connectivity. These included 17 protein-coding genes (circles) and 1 lncRNA (diamond). All of the hub genes, except for TSPAN3, were significantly over-expressed (FC: 1.5–2.0) among the RA_worsened_ women, compared to the RA_improved_. **B** Data on protein–protein interactions (PPI) from the STRING database showed that most of the protein-coding hub genes (14 of 18) encoded proteins that interacted with one another functionally

**Fig. 5:**
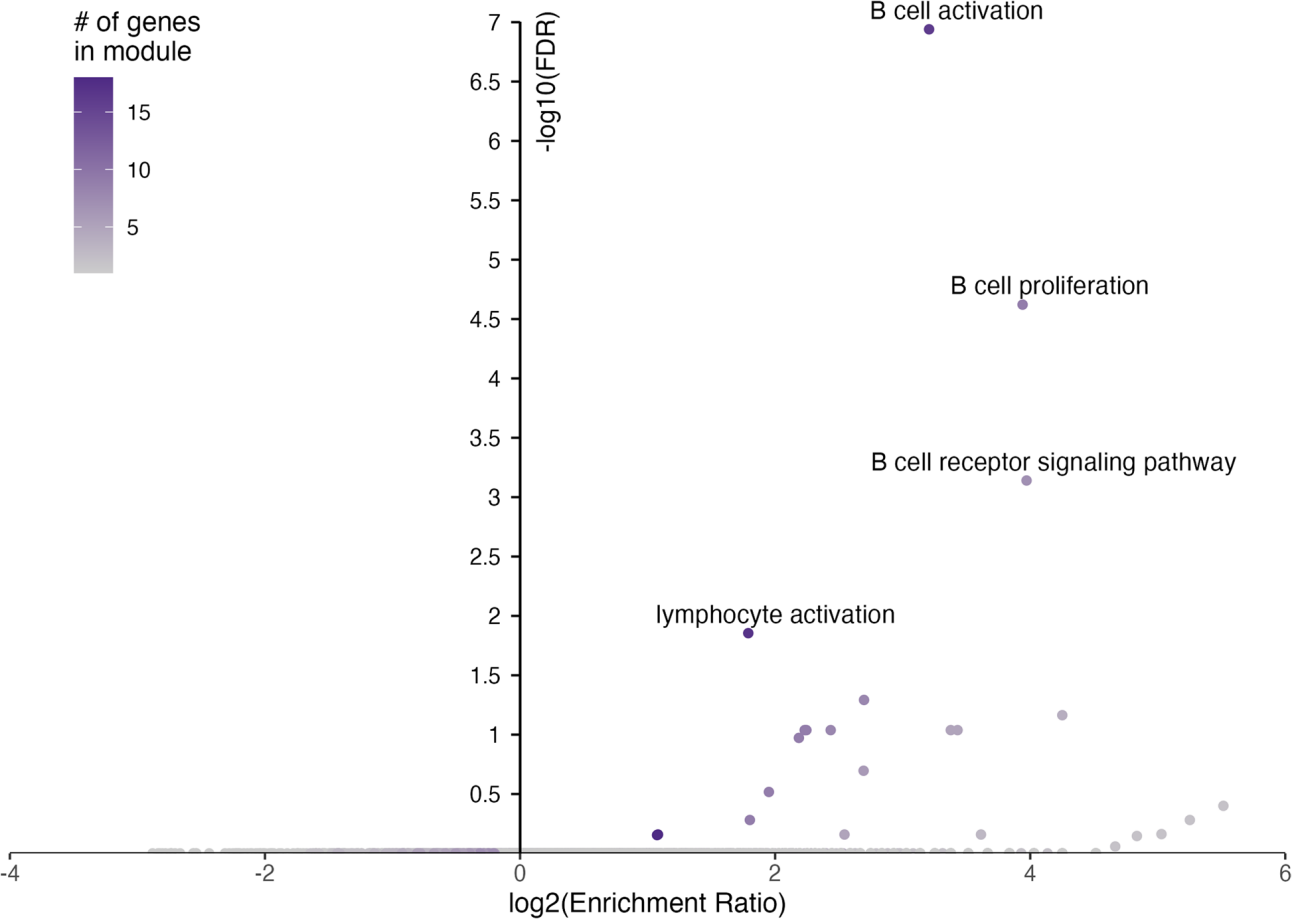
Gene Ontology (GO) terms enriched in genes that were co-expressed within the midnightblue module. Genes that were co-expressed within the midnightblue module were significantly enriched in GO terms related to B cell function

Within a WGCNA module, those genes with the highest degree of network connections—also known as hub genes—tend to have more biological relevance. In the midnightblue module, we identified 18 hub genes (17 protein-coding and 1 lncRNA) (Fig. 4A). Data on protein–protein interactions (PPI) from the STRING database showed that most of the protein-coding hub genes (14 of 18) encoded proteins that interacted with one another functionally (Fig. 4B). Furthermore, because genes clustered within a module are strongly co-expressed, the modules are often related to biological function. Functional enrichment analysis revealed that GO terms related to B cell function were enriched in genes co-expressed in the midnightblue module as well as in hub genes (Fig. 5). Interestingly, a significant correlation was observed between the midnightblue module eigengenes and our estimated proportions of naïve B cells in each of the RA_improved_, RA_worsened_, and healthy women at T0, with expression increasing as naïve B cell proportions increased (*r* = 0.8, FDR = 3E−07).

#### The salmon, light yellow, and blue modules

Of the 221 members of the salmon module, 50 were differentially expressed between the RA_improved_ and RA_worsened_ groups. These included CD40, IFI35, PARP10/12/14, SP140, STAT2, TAP2, and TLR7, among others. Genes in this module were functionally involved in GO terms such as innate immune response (FDR < 2.2E − 16) and type I interferon signaling pathway (FDR < 2.2E − 16). Of the 125 members of the light yellow module, 25 were differentially expressed between the RA_improved_ and RA_worsened_ groups. However, the light yellow module overall was not enriched in any specific GO terms. Additionally, while numerous neutrophil-related genes differentially expressed between the RA_improved_ and RA_worsened_ groups were co-expressed within the blue module, and the blue module eigengenes were under-expressed in the RA_worsened_ women, this module was not significantly associated with improvement or worsening of RA during pregnancy.

#### Transcription factor analysis

Transcription factor target enrichment analysis revealed that the genes that were co-expressed within the midnight blue module were significantly enriched among the target genes of the transcription factors PAX5, RFX5, GATA3, MEF2B, and RUNX3 (Additional file 4: Table S2). The genes that were co-expressed within the salmon module were significantly enriched among the target genes of the transcription factors STAT2, STAT1, IRF1, IRF2, IRF9, and SPIB.

## Discussion

In the present study, we used co-expression network analysis as a novel approach to examine pre-pregnancy transcriptomes from women with RA who subsequently improved or worsened during pregnancy in order to gain insight into the underlying biological differences. We show that, in our cohort of Danish women, a sub-network of highly co-expressed B cell-related genes was significantly correlated with the worsening of RA during pregnancy, while a sub-network of neutrophil-related genes was correlated with the improvement of RA. These findings are novel. There are no previous studies that have examined pre-pregnancy gene expression profiles in relation to subsequent pregnancy-induced improvement or worsening of RA, other than our previous report of differing RA-associated differential expression signatures between the two groups [3].

Although numerous genes were significantly differentially expressed between the RA_improved_ and RA_worsened_ women at T0, these findings relate to each gene independently of other genes, overlooking the fact that genes interact in complex biological networks. Co-expression network analysis, on the other hand, characterizes the correlation in the expression patterns among genes across samples in a dataset, clustering genes that are highly co-expressed within “modules.” In our data, of the 27 co-expression modules identified by WGCNA, only three (midnightblue, salmon, and light yellow) were both significantly enriched in genes that were differentially expressed between the RA_improved_ and RA_worsened_ women and were significantly correlated with our trait of interest, i.e., improvement/worsening during pregnancy. Furthermore, co-expressed genes within a module tend to be functionally related. Thus, the midnightblue and salmon modules were associated with GO terms related to B cell function and type I interferon signaling pathway, respectively, while the light yellow module was not functionally related to any specific GO term. In the midnightblue module, the large proportion of B cell-related genes (at least 44) among the differentially expressed protein-coding genes (*n* = 84) was striking. These B cell signature genes were involved in various B cell functions, such as antigen processing and presentation [HLA-DOA, HLA-DOB] [23], B cell receptor signaling [BANK1 [24], BLK [25], BLNK [26], CD19 [27], CD22 [28], CD72 [29], CD79A, CD79B [30], MS4A1 [31], NIBAN3 (also known as BCNP1) [32]], Fc receptors [FCRLA, FCRL1, FCRL2], transcription factor [E2F5 [33], EBF1 [34], LINC00926 [35], PAX5 [36], POU2AF1 [37], SPIB [38]], and other B cell functions including development, differentiation, migration, and others [COBLL1, CXCR5, FAM30A, TNFRSF13B]. Additionally, there were numerous genes of unknown function [e.g., AFF3, CORO2B, GNG7, OSBPL10, P2RX5, and SYNPO] that are known to be expressed in B cells based on expression data from The Human Protein Atlas [19] or from the validated B cell signature reported by Henning et. al. [21]. Furthermore, all of the midnightblue hub genes, which represent the genes with greater biological relevance within the module, have been involved in B cell function, including lncRNA LINC00926. Comparisons to healthy women revealed that the B cell signature was specific to the RA_worsened_ women. Thus, at the pre-pregnancy stage, the two groups of RA women differed significantly from each other in terms of B cell function.

We do not know at this stage if the observed pre-pregnancy B cell signature was due to the differences in the proportions of specific B cell sub-populations between the RA_improved_ and RA_worsened_ groups. Naïve B cells, memory B cells, and plasma cells were the only B cell sub-populations for which we could estimate cell proportions, and although naïve and memory B cell proportions were higher among the RA_worsened_ (vs RA_improved_) women, these differences were not statistically significant. Nevertheless, we found the estimated proportions of naïve B cells to be correlated with the midnightblue module eigengene expression, which suggests that naïve B cell proportions were contributing to the eigengene expression levels, which in turn were correlated with the worsening of RA during pregnancy. The involvement of naïve B cells in RA is supported by previous observations that activated autoreactive naïve B cells were present in the circulation of RA patients [39] and that naïve B cells were activated prior to an RA flare [40]. Hence, we speculate that the naïve B cells in the RA_worsened_ group may be autoreactive and that their proportion in the circulation may be a contributing factor in the worsening of RA during pregnancy.

Since co-expressed genes within a module tend to be co-regulated, another interesting finding was that 23 differentially expressed lncRNAs were co-expressed with the protein-coding genes within the midnightblue module, one of which was even identified as a hub gene. There is increasing evidence that lncRNAs may function as epigenetic regulators of immune-related gene expression in general and in RA [41, 42]. Co-expression analysis has previously been used to infer the function of some lncRNAs [43, 44], based on the premise that they would be functionally related to the protein-coding genes that they are co-expressed with (guilt-by-association), and whose expression they could potentially be regulating. In fact, some of the lncRNAs co-expressed in the midnightblue module have been previously associated with B cell function, and their increased expression have been associated with pro-inflammatory states. For example, hub gene LINC00926 has been implicated in regulating CD22 expression during B cell differentiation into plasma cells [35]. LINC00926 was co-expressed with TNFRSF13C and CD19 in breast cancer [45], as we observed in our data. In post-traumatic stress disorder (PTSD), increased expression of LINC00926 resulted in increased expression of pro-inflammatory cytokines [46]. Similarly, in a study of blood transcriptomes from vaccine cohorts, increased expression of lncRNA FAM30A in B cells was correlated with the expression of immunoglobulin (Ig) genes within the Ig heavy chain gene cluster on chromosome 14 (14q32.33) where FAM30A maps [47]. This supports our own observations that FAM30A was over-expressed (1.7-fold) in RA_worsened_ women, as were the immunoglobulin genes (1.6–1.9-fold). COPDA1, another lncRNA co-expressed in the midnightblue module, has been shown to upregulate the expression of MS4A1 [48], which encodes the B cell differentiation antigen CD20 [49]. In our data, lncRNA COPDA1 and MS4A1 were 3.7- and 1.8-fold over-expressed, respectively, in RA_worsened_ women. However, while anti-CD20 therapy (such as rituximab) has been used successfully to treat RA [50], it is not clear why CD20 expression was significantly higher in the RA_worsened_ group at the pre-pregnancy stage, when disease activity was similar to that in the RA_improved_ group. The co-expression of lncRNAs that have already been implicated in the regulation of expression of B cell-related genes, such as LINC00926, FAM30A, and COPDA, within the midnightblue module, and the enrichment of genes with midnightblue module membership among target genes for transcription factors such as PAX5, RFX5, MEF2B, RUNX3, and GATA3 suggest that at least a portion of the differentially expressed B cell-related genes are under transcriptional regulation. There is also a possibility that, for some genes, the differential expression could be due to a combination of differences in cell type proportions and transcriptional regulation.

The neutrophil signature observed among the RA_improved_ women at the T0 baseline was not as prominent as the B cell signature in the RA_worsened_ group. There was also no specific co-expression module in which the genes from the neutrophil signature were enriched, although some were co-expressed in the blue module. However, the expression patterns of genes in this module were not correlated with improvement or worsening during pregnancy.

The strengths of our study are as follows: Disease activity measures documented before and during pregnancy enabled us to assess clinical improvement or worsening of RA, based on MCID thresholds [9]. To our knowledge, this is the only pregnancy cohort that has clinical data from the same women both at a pre-pregnancy time point and during pregnancy, as well as biological samples available for gene expression assays. Furthermore, the homogeneous Danish background of the women in our cohort is advantageous in terms of minimizing heterogeneity in gene expression due to ethnicity. Using RNA-seq data from total RNA to evaluate gene expression enabled us to examine lncRNAs alongside the protein-coding RNAs. Our study also has limitations. First, sample sizes were small, especially for the RA_worsened_ group. Nonetheless, given the difficulties associated with identifying women with RA at the pre-pregnancy stage and given that there are no other such pre-pregnancy data available from women with RA, these pre-pregnancy gene expression data presented are unique and provide a valuable contribution to the field. Nonetheless, these findings need to be validated in an independent cohort of larger sample size. Second, because total RNA from the whole blood was used, the expression profiles of neutrophils could have dominated the observed expression patterns. Yet, although a neutrophil signature was observed among the RA_improved_ women, the sensitivity of RNA-seq technology enabled us to detect transcripts that were not neutrophil-specific, including a prominent B cell signature. Third, technical bias and/or batch effects could have been introduced in the data. However, we randomized sample order prior to sample processing, used a block design for sequencing, and at the data processing step, we used sample replicates to ensure that there were no batch effects. Fourth, we did not adjust for dosage and/or specific medications that may have an effect on the immune system due to the lack of variation in medication use within each of the RA groups. Additionally, although there is a possibility that changes in medications may have contributed to improvement or worsening during pregnancy, that is not entirely clear given that two women who discontinued Infliximab and all medication, respectively, improved during pregnancy, while another woman worsened despite maintaining biologic therapy throughout pregnancy.

## Conclusions

In our Danish pregnancy cohort, differential gene expression analysis showed little overlap in RA-associated pre-pregnancy (T0) gene expression signatures between the RA_improved_ and RA_worsened_ women, suggesting that the two groups of women differ significantly in their T0 expression profiles. Using co-expression gene network analysis as a system-based approach to build on the differential expression data, we identified a co-expression module related to B cell function that was correlated with subsequent worsening of RA during pregnancy. This module was also significantly enriched in a set of B cell-related genes that were differentially expressed between the RA_improved_ and RA_worsened_ groups at T0. Additionally, several neutrophil-related genes were significantly over-expressed among the RA_improved_ women at the T0 baseline. These pre-pregnancy expression signatures associated with the subsequent improvement or worsening of RA during pregnancy represent potential predictive biomarkers that could have important implications in terms of a personalized approach to the treatment of RA during pregnancy.

### Supplementary Information

**Additional file 1: Fig. S1.** PCA plot of normalized counts for quality control. Following rigorous quality control of the data, log_2_-transformed TMM-normalized counts data (CPM) from all genes were plotted as a Principal Components Analysis (PCA) plot for pre-pregnancy (T0) samples from 14 RA women who subsequently improved during pregnancy, 5 who worsened and 13 healthy women.

**Additional file 2:** **Fig. S2.** Relative proportions of different cell populations at the pre-pregnancy baseline among the RA_improved_, RA_worsened_ and healthy women. The box plots show how the relative proportions of different cell types estimated using CIBERSORTx compared between the RA_improved_, RA_worsened_ and healthy women at the pre-pregnancy (T0) baseline. Only cell types included in the LM22 reference dataset are shown. For some LM22 cell types, proportion estimates were not obtained from CIBERSORTx; those are not shown here (resting dendritic cells, activated mast cells, activated NK cells, gamma delta T cells).

**Additional file 3: Table S1.** All protein-coding genes, lncRNAs and miRNAs analyzed for differential expression between the RA_improved_ and RA_worsened_ groups (n=19,468) are included in this table showing the output from the analysis. logFC refers to the log(fold-change) in the RA_worsened_ compared to the RA_improved_ group. Information on novel lncRNAs identified in our data (names starting with MSTRG) and included in the analyses are available upon request.

**Additional file 4:** **Table S2.** Transcription factor target genes co-expressed within the midnightblue and salmon modules. Several of the genes that were co-expressed within the midnightblue and salmon modules were identified as target genes for transcription factors. Only transcription factors whose target genes were significantly enriched in co-expressed genes within each module are shown. Target genes in bold represent genes that were also differentially expressed between the RA_improved_ and RA_worsened_ women.

## Data Availability

The datasets presented in this article are not readily available because the data and materials are protected by the General Data Protection Regulation (GDPR) of the European Union (2016/679) and by the Danish Data Protection Act enacted in May 2018 to supplement the GDPR. However, the authors commit to making the relevant anonymized expression level data available on reasonable request. Such requests should be directed to the corresponding author, Dr. Damini Jawaheer.

## Abbreviations

CDAI: Clinical Disease Activity Index
CRP: C-reactive protein
FC: Fold change
FDR: False discovery rate
GLM: Generalized linear model
GO: Gene Ontology
lncRNAs: Long non-coding RNAs
MCID: Minimum clinically important difference
PC: Principal components
PCA: Principal component analysis
PPI: Protein-protein interaction
RNA-seq: RNA sequencing
RA: Rheumatoid arthritis
RA_improved_: Women with RA who subsequently improved during pregnancy
RA_worsened_: Women with RA who subsequently worsened during pregnancy
STRING: Search Tool for the Retrieval of Interacting Genes
T0: Pre-pregnancy
T3: Third trimester
TF: Transcription factor
TMM: Trimmed mean of *M*-values
WGCNA: Weighted gene co-expression network analysis
