# Supplementary figures and images for: Pre-pregnancy gene expression signatures are associated with subsequent improvement/worsening of rheumatoid arthritis during pregnancy
Source: Arthritis Res Ther. 2023 Oct 4;25:191. doi: 10.1186/s13075-023-03169-6 (PMC10548620; doi:10.1186/s13075-023-03169-6)

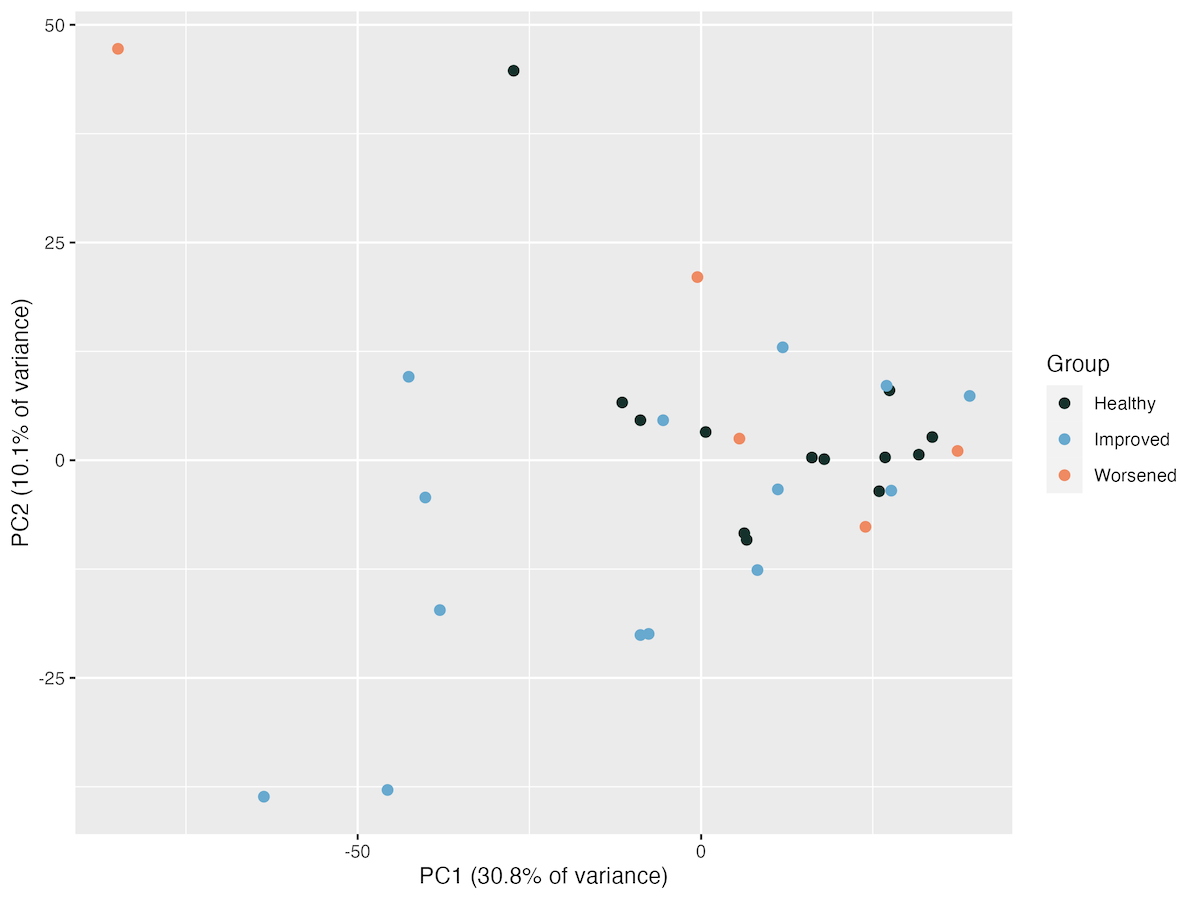

Supplement: Supplementary file 1 — Additional file 1: Fig. S1. PCA plot of normalized counts for quality control. Following rigorous quality control of the data, log2-transformed TMM-normalized counts data (CPM) from all genes were plotted as a Principal Components Analysis (PCA) plot for pre-pregnancy (T0) samples from 14 RA women who subsequently improved during pregnancy, 5 who worsened and 13 healthy women. [file 13075_2023_3169_MOESM1_ESM.tiff]

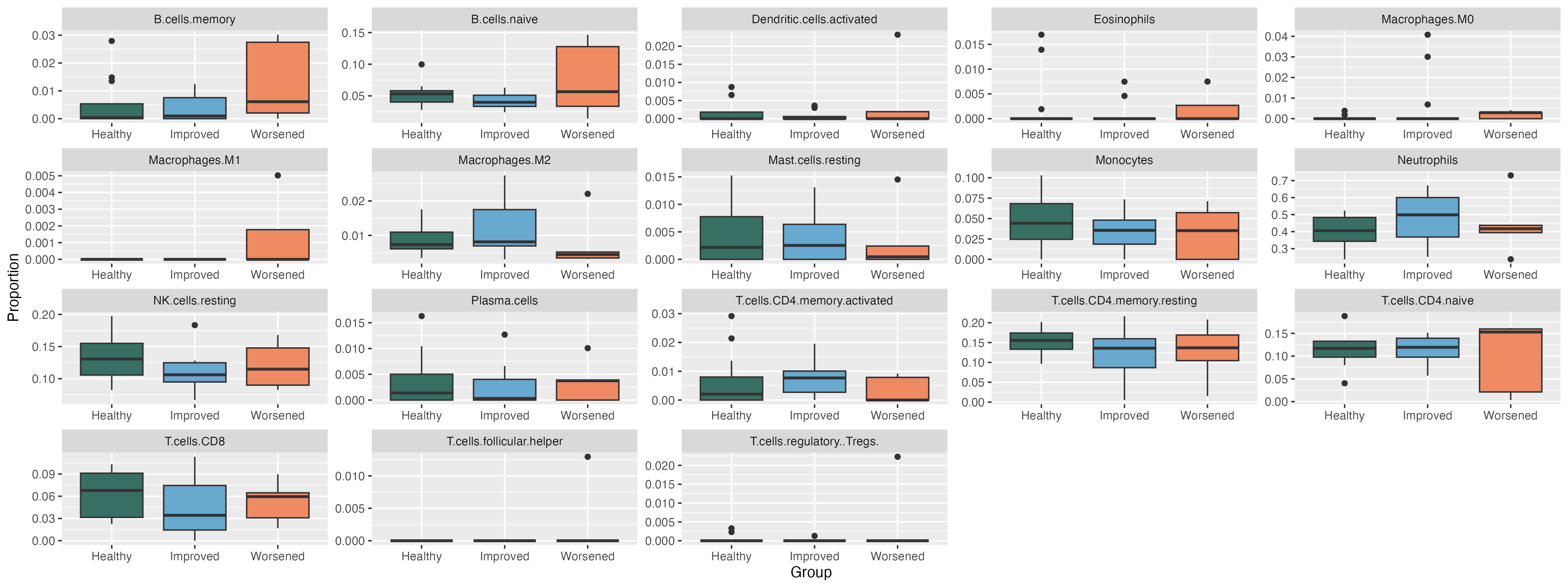

Supplement: Supplementary file 2 — Additional file 2: Fig. S2. Relative proportions of different cell populations at the pre-pregnancy baseline among the RAimproved, RAworsened and healthy women. The box plots show how the relative proportions of different cell types estimated using CIBERSORTx compared between the RAimproved, RAworsened and healthy women at the pre-pregnancy (T0) baseline. Only cell types included in the LM22 reference dataset are shown. For some LM22 cell types, proportion estimates were not obtained from CIBERSORTx; those are not shown here (resting dendritic cells, activated mast cells, activated NK cells, gamma delta T cells). [file 13075_2023_3169_MOESM2_ESM.tiff]
