# Supplementary material for: Pre-pregnancy gene expression signatures are associated with subsequent improvement/worsening of rheumatoid arthritis during pregnancy
Source: Arthritis Res Ther. 2023 Oct 4;25:191. doi: 10.1186/s13075-023-03169-6 (PMC10548620; doi:10.1186/s13075-023-03169-6)
Supplement: Supplementary file 4 — Additional file 4: Table S2. Transcription factor target genes co-expressed within the midnightblue and salmon modules. Several of the genes that were co-expressed within the midnightblue and salmon modules were identified as target genes for transcription factors. Only transcription factors whose target genes were significantly enriched in co-expressed genes within each module are shown. Target genes in bold represent genes that were also differentially expressed between the RAimproved and RAworsened women. [file 13075_2023_3169_MOESM4_ESM.pdf]

Table S2. Transcription factor target genes co-expressed within the midnightblue and salmon modules

Several of the genes that were co-expressed within the midnightblue and salmon modules were identified as target genes for transcription factors. Only transcription factors whose target genes were significantly enriched in co-expressed genes within each module are shown. Target genes in bold represent genes that were also differentially expressed between the $RA_{improved}$ and $RA_{worsened}$ women.

Transcription factor	Module genes enriched among target genes for transcription factor	Number of known target genes	Overlap	Fold enrichment	FDR
**midnightblue module**					
PAX5	BCL11A, **BLK**, **CD19**, **CD22**, **CD79A**, FCER2, MACF1, **POU2AF1**	55	8	16	1.1E-05
RFX5	**HLA-DOA**, **HLA-DOB**, MACF1, **NXPH4**, SYBU, TSPAN3	85	6	8	2.0E-02
GATA3	**AFF3**, ANKRD28, **BANK1**, **CCSER1**, **CHL1**, **EBF1**, **IL7**, LAMC1, **MACROD2**, **OSBPL10**, **PMEPA1**, **PTPRK**, RAB30, SYBU, **TCF4**, TPD52	635	16	3	2.0E-02
MEF2B	**BLK**, **E2F5**, **KCNH8**, MACF1	33	4	14	2.0E-02
RUNX3	BCL11A, **PAX5**, **AFF3**, **BLK**, **POU2AF1**, **TCF4**	100	6	7	2.1E-02

**salmon module**					
STAT2	**STAT1**, APOL1, **APOL6**, BST2, CMPK2, **CMTR1**, **DBF4B**, DDX58, DDX60, DDX60L, **DHX58**, DRAP1, DTX3L, EPSTI1, FAM111A, **IFI35**, IFI6, IFIH1, IFIT1, IFIT3, IFIT5, IFITM1, IRF9, ISG15, LAP3, **MOV10**, MX1, OAS1, OAS2, OAS3, OASL, **PARP10**, **PARP12**, **PARP14**, PARP9, PHF11, **PML**, **RNF213**, RTP4, SAMD9L, SP110, **TAP2**, **TRIM14**, TRIM22, UBE2L6, XAF1, **ZC3HAV1**	104	47	40	5.0E-62
STAT1	AFF1, **APOL6**, BST2, **CD40**, CXCL10, DTNBP1, EPB41L3, **ETV7**, **GBP1**, **GRAMD1B**, HIVEP2, **IFI35**, IFI6, IFIT1, IFIT2, IFIT3, IFITM1, IFITM3, IRF9, ISG15, LAP3, LHFPL2, MOB3C, MX1, MYOF, OAS1, OAS2, OAS3, OASL, **PARP14**, PLSCR1, **PML**, **RHBDF2**, **RNF213**, RSAD2, **SAMD4A**, **SHFL**, SLC12A8, SP110, **STAT2**, TCF7L2, TNFSF13B, **TTC26**, **ZC3HAV1**, ZCCHC2	1001	45	4	2.7E-13
IRF1	AFF1, **CD40**, CXCL10, DDX58, DDX60L, IFIT2, IFIT3, ISG15, STAT1, **TAP2**, TCF7L2, UBE2L6, **ZC3HAV1**	76	13	15	3.9E-10
IRF9	DLGAP1, IFIT1, IFIT3, ISG15, MX1, OAS2, OAS3, **PML**, RSAD2, SAMD9L	47	10	19	1.0E-08
IRF2	AFF1, BTN3A1, FAM111A, **FANCA**, IFIT3, ISG15, MICB, **NUB1**, **PARP14**, UBE2L6, **ZC3HAV1**	76	11	13	7.4E-08
SPIB	**CD40**, DLGAP1, PDCD1LG2, SP110, **SP140**	58	5	8	3.3E-02
